# Genomic insights and functional adaptation of *Mycobacterium intracellulare* strains isolated from patients with inflammatory bowel disease

**DOI:** 10.1128/spectrum.03414-24

**Published:** 2025-10-20

**Authors:** Sushanta Deb, Lata Kumari, Neeraj Mahajan, Shiv K. Basant, Twinkle Joshi, Vineet Ahuja, Urvashi B. Singh

**Affiliations:** 1 Department of Microbiology, AIIMS 233643, New Delhi, India; 2 Department of Gastroenterology, AIIMS, New Delhi, India; ARUP Laboratories, Salt Lake City, Utah, USA

**Keywords:** *Mycobacterium*, phylogenomics, genomics, next-generation sequencing

## Abstract

In clinical practice, inappropriate diagnosis of inflammatory bowel disease (IBD) ascribing the disease to *Mycobacterium avium paratuberculosis* (MAP) and resulting mistreatment has been a challenge for healthcare workers. This highlights the need to accurately identify and characterize MAP members in patients with gastrointestinal (GI) ulceration. A total of 889 patients exhibiting symptoms of IBD were examined for MAP infection. While MAP was not recovered from any of the specimens, we successfully isolated and sequenced four clinically relevant *Mycobacterium intracellulare* genomes from GI samples of patients diagnosed with IBD. In-depth phylogenomic and comparative genomics demonstrated genomic heterogeneity and variable metabolic profile in *M. intracellulare* species. Analysis of four isolate genomes disclosed an ongoing genome reduction process, which might be crucial for adaptation and increased virulence of intestinal *M. intracellulare* strains. Findings provide insights into the narrow-spectrum resistance to antibiotics, metabolic adaptation, and potential virulence of *M. intracellulare* strains, particularly those associated with human infections. Recombination analysis revealed minimal recombination and higher mutational divergence, which indicate evolutionary constraints leading to clonal evolution of *M. intracellulare* species.

**IMPORTANCE:**

The present study provides valuable insights on genomic diversity, metabolic adaptability, and virulence of *Mycobacterium intracellulare* strains infecting human host. Our findings highlight the need for additional research on essential genes as potential drug targets. An updated knowledge on intestinal *M. intracellulare* strains regarding its genomic characteristics and evolutionary processes is critical to design better diagnostic and treatment approaches for intestinal infections.

## INTRODUCTION

Mycobacterial diseases have a millennium-long history and continue to pose significant health challenges in both developed and developing regions worldwide (1). Next-generation sequencing (NGS) and comparative genomic approaches facilitated robust and distinctive classifications of Mycobacterium members. Comparative genomic tools, combined with complex bioinformatics algorithms, have facilitated in-depth exploration of genomic and evolutionary signatures, providing high-resolution accuracy in the identification of *Mycobacterium avium* complex (MAC) members (2, 3). *M. avium* and *M. intracellulare* are key MAC members, ubiquitously present in soil, livestock populations, and aquatic ecosystems. Their ability to survive intracellularly makes them suitable candidates for affecting body organs and gastrointestinal (GI) tract. *M. intracellulare*, involved in disseminated and localized GI infections, poses a higher risk for individuals with compromised immune systems (4). Earlier genomic studies reported multiple clinically important *M. intracellulare* strains, primarily isolated from patients identified with pulmonary disease (5–7). A recent study by Jia et al. employed a combination of comparative genomics and machine learning techniques to identify specific diagnostic markers crucial to distinguish between various pathogenic non-tuberculous mycobacteria, with a particular emphasis on the *M. intracellulare* species (8). Furthermore, Hirama et al. (9) show the importance of *M. intracellulare*, highlighting its clinical relevance and frequent isolations from immunocompromised patients (9). Exploration of genetic diversity and evolutionary trends could offer valuable insights into adaptive mechanisms and pathogenic potential of *M. intracellulare* species group (10). *M. intracellulare* and its subspecies *M. intracellulare* subsp. *chimaera* have been associated with human infections, the former involved in pulmonary infections, while the latter conclusively linked to fatal infections following cardiac surgery (11, 12). Two *M. intracellulare subsp.,* which includes *M. intracellulare subsp. yongonense* and *M. intracellulare* subsp*. chimaera* were reclassified from *M. yongonense* and *M. chimaera*, respectively (13). The marked increase in the incidence of *M. intracellulare* infections in recent years raised significant concern and attention (14). Therefore, proper identification and timely treatment are essential to manage and control the *M. intracellulare* infection risk in immunocompromised patients. To date, considerable whole genome reports of *M. intracellulare* are available, and they are primarily isolated from lung tissue of patients with pulmonary MAC disease (13, 15). Intestinal *M. intracellulare* is highly underexplored; case studies reporting gut-dwelling *M. avium intracellulare* are very rare (4, 16). As per current knowledge, the complete genome sequence of *M. intracellulare* isolated from the human GI tract remains unreported. This is the first whole-genome sequencing (WGS) report of intestinal *M. intracellulare* strains isolated from human gut. Here, we performed comprehensive phylogenomic and comparative genomic analysis to determine distinctive genomic features of four intestinal *M. intracellulare* isolates. This study observed distinctive genomic features among *M. intracellulare* isolates through comparison with other publicly available non-intestinal *M. intracellulare* genomes. The study highlights the potential involvement of reductive evolution (17) and the loss of functionally essential genes (18) in adaptation of intestinal *M. intracellulare* strain. Recombination, resistance, and virulence gene analysis reveal large-scale genomic heterogeneity within the members of *M. intracellulare* species. Understanding this information is essential for developing advanced treatments and management strategies to mitigate the risk of *M. intracellulare* infection.

## MATERIALS AND METHODS

Colonic biopsy and blood buffy coat samples were processed for cultures and DNA detection for mycobacterial pathogens (including IS900 sequences highly specific to MAP) in 889 patients with inflammatory bowel disease (IBD); Crohn’s disease (CD) (clinical manifestations, endoscopic, radiological, and histological features as per ECCO consensus guidelines), Intestinal tuberculosis (clinical, radiological, endoscopic, histopathological features), Ulcerative colitis (as per ECCO consensus guidelines), and Controls (Adult patients with hemorrhoidal bleeding and no other colonic disease undergoing sigmoidoscopy).

### Clinical observations

#### IBD 3019 (isolate A1): 32-year-old male patient with ulcerative colitis

Staying in a rural area and a vegetarian, non-smoker, non-alcohol consumer, with history of consumption of non-pasteurized milk, Modified Kuppuswami SES scale of 16 with symptoms duration of 24 months prior to diagnosis, disease extent of Left-sided colitis (Montreal Classification: E2), and mild disease activity, was started on oral 5 amino salicylates (5ASA) and has remained in remission until last follow-up.

#### ITB 2346 (isolate B1): 30-year-old male patient with intestinal TB

Staying in a rural area and a non-vegetarian, current smoker, non-alcohol consumer, with history of consumption of non-pasteurized milk, Modified Kuppuswami SES scale of 10 with symptoms duration of 12 months prior to diagnosis, with an inflammatory phenotype and involvement of terminal ileum (Montreal classification A2B1L1), with mild disease activity. Ileal biopsy sent for MTB and MAP culture. Subsequently, given anti-tubercular therapy and showed a complete symptomatic response and mucosal healing.

#### IBD3273 (isolate C1): 19-year-old male patient with ulcerative colitis

Staying in a rural area and a vegetarian, non-smoker, non-alcohol consumer, with history of consumption of non-pasteurized milk, Modified Kuppuswami SES scale of 8 with symptoms duration of 6 months prior to diagnosis, with disease extent of left-sided colitis (Montreal Classification: E2) with moderate activity and was given oral steroids and 5ASA. Thereafter, he remained in remission on 5ASA for the next 3 years (until last follow-up).

#### ITB 2409 (isolate S24): 23-year-old male patient with intestinal TB

Staying in a rural area and a non-vegetarian, non-smoker, non-alcohol consumer, with a history of consumption of non-pasteurized milk, Modified Kuppuswami SES scale of 8 with symptoms duration of 24 months prior to diagnosis, with an inflammatory phenotype and involvement of terminal ileum (Montreal classification A2B1L1), with mild disease activity. Ileal biopsy sent for MTB and MAP culture. Subsequently, given anti-tubercular therapy and showed a complete symptomatic response and mucosal healing.

### Microbial culturing

The laboratory was blinded to the clinical presentation and supporting findings of the patients. The processed samples were subjected simultaneously to AFB smear, liquid culture, and GeneXpert MTB/RIF (to rule out tuberculosis). Colonic biopsy and blood buffy coat samples were subjected to standard N-acetyl-l-cysteine sodium hydroxide (NALC-NaOH) decontamination and concentration by centrifugation. The concentrated sediment from each sample was cultured on two MGIT tubes (OADC, PANTA, and 2 µg Mycobactin J) to rule out *M. tuberculosis/M. bovis* and incubated at 37°C. Briefly, the sediment was transferred to the tube containing 4.5 mL of MGIT medium (Becton Dickinson) supplemented with 10% oleic acid-albumin-dextrose-catalase (OADC), PANTA (40 U of polymyxin B per mL, 4 µg of amphotericin B per mL, 16 µg of nalidixic acid per mL, 4 µg of trimethoprim per mL, 4 µg of azlocillin per mL [final concentrations]), and 2 µg of mycobactin J (Allied Monitor, Fayette, MO) per mL. The cultures were incubated for 8 weeks and examined weekly for evidence of growth. For MAP, the cultures were incubated for 1 year with IS 900 PCR performed every month, as the MGIT 960 may not give a positive signal. 0.5 mL culture was removed aseptically for testing by the IS*900*-specific nested PCR as described below. In order to rule out contamination, when the MGIT vial gives a positive reading, 0.1 mL of vial contents was plated on blood agar/Mueller Hinton Agar and a smear stained by both Gram’s and acid-fast methods examined by light microscopy. Contaminants were characterized only by colonial and cellular morphology and Gram stain reaction. Mycobacterial isolates other than *M. avium* subsp. *paratuberculosis* were identified by standard methods.

### WGS, assembly, and annotation: genomic DNA extraction and WGS

*Mycobacterium* isolates were cultured in Lowenstein-Jensen medium and incubated at 37°C. Bacterial cultures were suspended into 0.1 mL PBS and heat-inactivated at 95°C for 40 min. Heat-inactivated samples were transferred to BSL2 lab, added 40 µL of lysozyme, and incubated at 37°C for 2 h in shaking water bath. After incubation, 56 µL of 10% SDS and 5 µL of proteinase K (20 mg/mL) were added, vortexed, and incubated at 65°C for 30 min. To this 80 µL of 5 M NaCl; 64 µL of CTAB-NaCl was added, vortexed, and incubated at 65°C for another 30 min. Genomic DNA was purified with standard chloroform-isoamyl alcohol DNA extraction procedure.

Purified DNA was analyzed with Qubit 4 Fluorometer (Thermo Scientific) and agarose gel electrophoresis. The library was prepared by Illumina DNA Prep workflow using Nextera DNA Flex library prep kit as per the manufacturer’s instructions (Illumina Inc., USA). Briefly, the DNA was subjected to tagmentation, post-tagmentation cleanup, amplification of tagmented DNA, and library cleanup. The libraries were then analyzed using Qubit 4 Fluorometer (Thermo Fisher Scientific) and Fragment Analyzer (Agilent Technologies, Inc., USA). The libraries were then normalized using re-suspension buffer and pooled to a final starting concentration of 4 nM. The pooled 4 nM libraries were then denatured using freshly prepared 0.2 N NaOH. The denatured libraries were then diluted to a final loading concentration of 12 pM. A total volume of 600 µL was loaded onto the MiSeq instrument using MiSeq Reagent kit v3 (600 cycles).

### Assembly and annotation

To assess the quality and contamination of Illumina reads, we employed the FastQC v0.11.5 tool. In order to ensure the accuracy of the genetic data, we utilized the Trimmomatic v0.36 tool in default settings to remove any low-quality reads and adapter sequences (19). We employed Unicycler v.0.4.7 (20) in default mode to assemble the trimmed reads *de novo*, followed by quality assessment of assembled genomes using QUAST 4.6.1 (21). The assembled genomes were annotated using the NCBI Prokaryotic Genome Annotation Pipeline (PGAP version 4.9) with default parameters (22). To ensure the accuracy, we evaluated the completeness and contamination of genomes using CheckM2 tool (23). Assembled genomes were taxonomically assigned at the species level using GTDB-tk V2 program (24).

### Comparative genomics

The genomic sequences of *M. intracellulare* strains were retrieved from the NCBI genebank using NCBI-genome-download (https://github.com/kblin/ncbi-genome-download) program and assessed their quality using the CheckM2 tool (23). To determine species boundaries, overall genome relatedness indices (OGRIs) specifically ANIb, AAI, and *in silico* DDH (*ins*DDH) values were obtained, and genomic relatedness of four isolates was estimated against reference *M. intracellulare* ATCC 13950T strain (25). To construct a core genome tree, genes were selected based on a BlastP search (*e*-value = 1.0 e−15; alignment coverage of ≥70%) among *M. intracellulare* genomes (26, 27). Recombinant genes were removed from downstream analysis with the help of the PhiTest feature implemented in the PhiPack program (28). The identified core genes, deemed statistically non-recombinant, were concatenated, aligned, and used to construct a maximum-likelihood phylogeny with Generalized Time-Reversible model using the RAxML tool (29, 30). Virulence genes were identified using ABRicate software (https://github.com/tseemann/abricate), using blast search against two databases—VFDB 2022 (31) and MEGARes 2.0 (32) with a 90% threshold coverage and identity. In order to investigate antimicrobial resistance genes, the genomes of *M. intracellulare* strains were subjected to blast screening using two databases: the Comprehensive Antimicrobial Resistance Database (33) and the RESfinder (34).

The clusters of orthologous groups (COG) functional annotation of coding sequences obtained through RPS BLAST search against COG database (https://ftp.ncbi.nih.gov/pub/COG/) with the help of Perl script CDD2COG.pl v0.2 (https://github.com/aleimba/bac-genomics-scripts/tree/master/cdd2cog). A heatmap generated using the Manhattan distance and average clustering method embedded in the heatmap2 function of the gplots package in the R program to illustrate the clustering of genomes based on the distribution of genes across various COG categories. We compared recombination parameters and calculated correlation profiles among *M. intracellulare* genomes. We used the mcorr tool to measure the degree of correlation between any two loci in the core genome separated by a defined number of nucleotides (35). This allowed us to calculate five recombination parameters: sample diversity, recombination coverage, mutational divergence, recombinational divergence, and relative rate of recombination to mutation (35). In a subsequent analysis, crucial genomic regions that are fundamental and non-essential have also been identified (36). DELEAT confidently employs a logistic regression classifier that has been trained on a highly selective subset of organisms from the Database of Essential Genes (36). This classifier assigns an essentiality score to every gene in a genome, with an essentiality threshold of 0.75, enabling the identification of essential and non-essential regions with precision (36). The score, which ranges from 0 to 1, is based on multiple gene features and represents the gene’s probability of belonging to the class “essential.” This tool is also proficient in designing large-scale dispensable regions across bacterial genomes, guided by two parameters: the minimum desired deletion length (L) and the essentiality score (E) (36). A summary is generated which includes factors such as deletion size, the count of deletions, and the order of deletions within four *M. intracelluare* genomes sequenced in this study. Roary version 3.11.2 in default setting used to analyze *M. intracellulare* genomes in gff format, and pan-genome plots were generated using create_pan_genome_plots.R (37). Furthermore, the PIRATE (v.1.0.3) pangenomics tool was used to identify unique alleles present in at least one genome and cluster orthologous genes present in diverse genomes, which facilitates the distinction of closely related *M. intracellulare* strains (38). This study performed a genome-wide mapping between reference *M. intracellulare* genomes and intestinal *M. intracellulare* isolates and visualized through pairwise comparison using BLASTP searches in the CGView Comparison Tool to identify regions of differences (RODs).

## RESULTS

### General genomic feature of *M. intracellulare* species

Four *M. intracellulare* isolate (A1, B1, C1, and S24) genomes were sequenced and annotated using the NCBI PGAP pipeline. The general genomic features and OGRI values are listed in Table 1. A total of 82 *M*. *intracellulare* genomes were obtained from the NCBI database using the getSequenceInfo tool (https://github.com/dcouvin/getSequenceInfo). The genomes were sorted according to their isolation source, genome sizes, and GC contents ranged from 5.2 to 6.6 Mbases and from 67.2% to 68.6%, respectively (see Table S1 at https://doi.org/10.6084/m9.figshare.30356617.v1). ANI and AAI values were higher than the established threshold values of 95–96%, which indicates isolates belong to the *M. intracellulare* species (39, 40). In addition, *in silico* genomic DNA–DNA hybridization (*ins*DDH) values surpassed the 70% cutoff criterion, further validating the species demarcation of the sequenced genomes as *M. intracellulare* (41). Altogether, the ANI, AAI, and insDDH data collectively support the classification of all four isolate genomes as *M. intracellulare* species (Table 1).

**TABLE 1:** Genome statistics and OGRI values of four *M. intracellulare* isolates (values obtained comparing isolate genome against reference *M. intracellulare* ATCC 13950T strain)

Strain	Accession no.	CDS	G+C content (%)	No. of contigs	Genome size (bp)	Coverage depth	ANIb (%)	*In silico* DDH	AAI (%)	Completeness	Contamination
A1	JAUZTK010000000	5,147	67.89	150	5485955	42.6×	96.95	80.10%	97.97%	99.56	0.72
B1	JAUZTJ010000000	5,120	67.98	115	5449394	45.1×	97.46	83.20%	98.47%	99.94	0.79
C1	JAUZTI010000000	5,045	67.84	294	5233020	34.6×	96.71	80.10%	97.86%	85.88	2.71
S24	JAKFBO010000000	5,441	67.8	157	5810383	43.6×	96.92	78.50%	97.77%	99.44	0.19

### Core genome phylogeny and functional profiling of *M. intracellulare* strains

**Fig 1:**
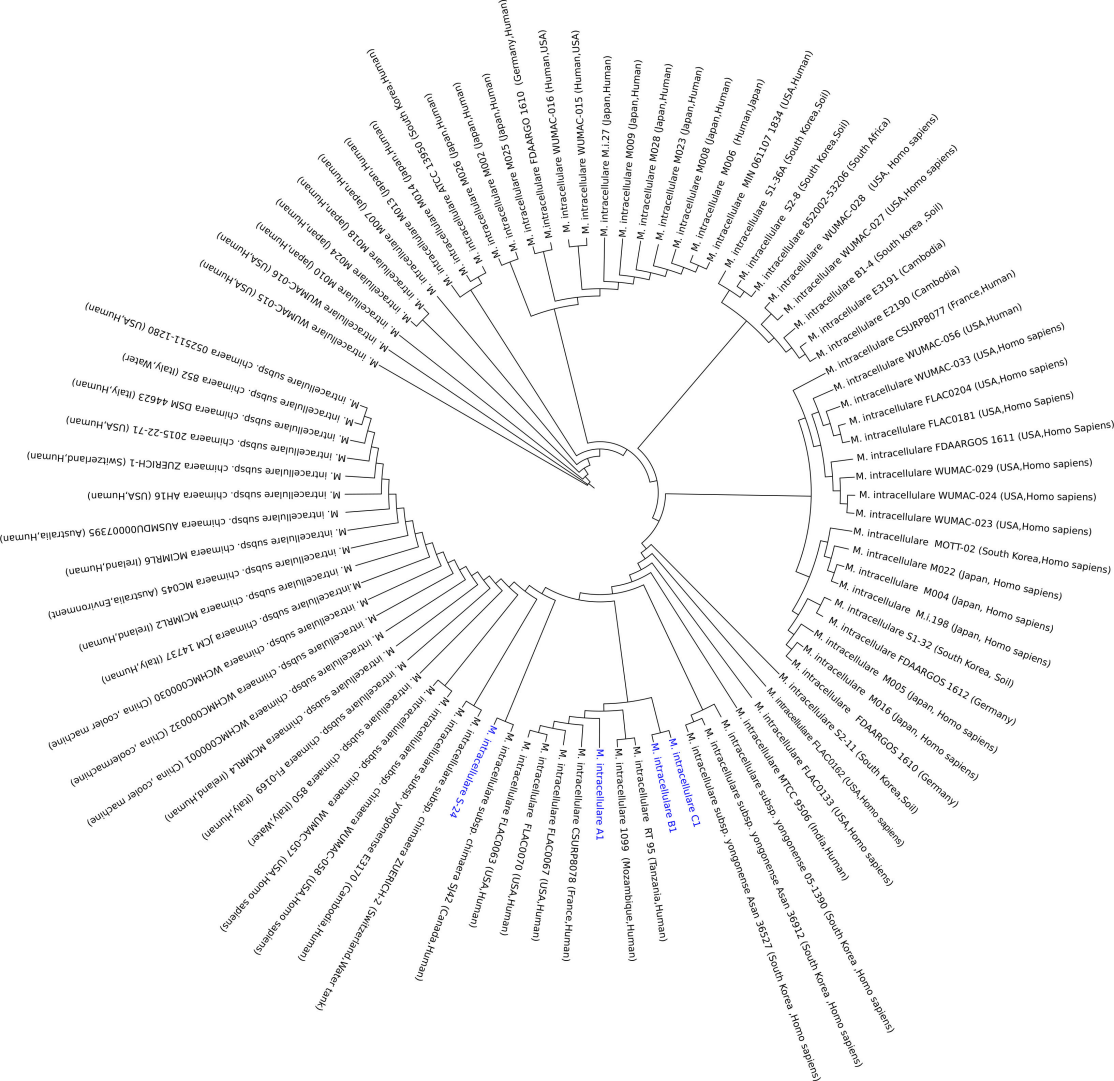
Maximum-likelihood core genome phylogenomic tree of the four intestinal isolate *M. intracellulare* genomes (colored in blue) and 82 publicly available *M. intracellulare* genomes (colored in black). This tree is derived from the concatenated alignment of 1,824 core genes, ensuring a robust phylogenetic reconstruction. Geographical locations and isolation sources of the genomes are annotated on tips of the tree.

Core genome phylogeny reconstruction has been frequently used for evolutionary analyses (42), host-specificity and outbreak surveillance owing to various pathogens, such as *Mycobacterium tuberculosis* (43), *Pseudomonas aeruginosa* (44), and *Listeria monocytogenes* (45). The gene-by-gene comparative approach identifies core genes and leverages them to deduce evolutionary relationships among the members of a given species (46). To investigate the phylogenomic relationships among *M. intracellulare* strains, we construct a maximum likelihood phylogenetic tree using the non-recombinant core genes (1,824 genes) present in all *M. intracellulare* genomes (86 genomes). A close look into the core gene tree infers a weak association between the *M. intracellulare* strains and their geographical isolation source. The core gene phylogeny of *M. intracellulare* genomes does not form a distinct clade and shows no clustering based on geographical location or host ecology. Although few phylogenetic clades are distinct to US and Japan isolates, this is mainly due to the clonal strains isolated from a local region (Fig. 1). S-24 strain showed distant phylogeny, while A1, B1, and C1 clustered in a single clade sharing a recent common ancestor, which was possibly due to the high genetic diversity of S-24 strain (Fig. 1). Furthermore, the phylogenetic position indicates S-24 close relatives are primarily subspecies of *M. intracellulare*, which supports the notion that S-24 isolate may belong to the *M. intracellulare* subspecies group (Fig. 1). These members exhibit complex evolutionary signals and high genetic heterogeneity, leading to frequent sub-cladding in the phylogenetic tree (multiple descendant lineages) (Fig. 1).

**Fig 2:**
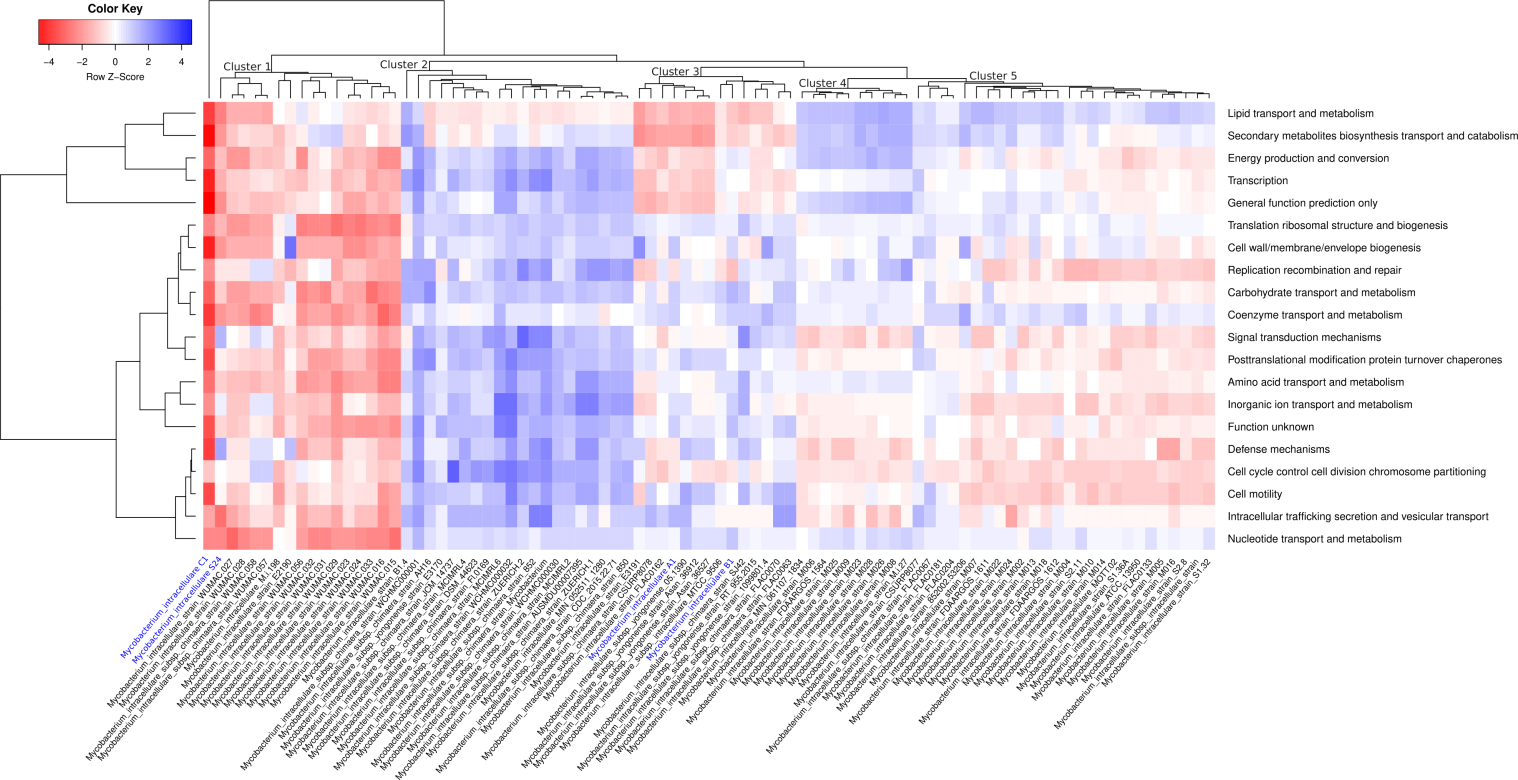
Distribution of COG metabolic function across *M. intracellulare* genomes. Differentially enriched metabolic functions are presented for isolate *M. intracellulare* genomes (colored in blue) and publicly available *M. intracellulare* genomes (colored in black). Raw *z*-score (scale range from −4 to +4) indicates abundance of genes associated with each functional category illustrated with red (low abundance) and blue (high abundance) colors. The hierarchical clustering of genomes was performed using a weighted Bray-Curtis approach.

Here, we also have investigated genome-encoded metabolic functions to provide valuable insights into the distinct functional profile of each *M. intracellulare* strain. Hierarchical clustering of *M. intracellulare* genomes based on COG function categories demonstrated high variability in the relative enrichment of metabolic functions. The COG functional heatmap shows clear grouping of *M. intracellulare* strains based on their metabolic profile. We observed five distinct clusters with varied metabolic profiles (Fig. 2). However, isolates C1 and S-24 (from this study) exhibited highly reduced metabolic capability, and few genes were assigned to any metabolic function (Fig. 2). This reduced metabolic function observed in the C1 strain may be attributed to poor sequencing or assembly quality, as indicated by low completeness and high contamination levels (Table 1). Clusters 1 and 5 exhibited a reduced metabolic potential relative to other *M. intracellulare* strains (Fig. 2).

### Genome mapping identifies ROD in four *M. intracellulare* isolates

**Fig 3:**
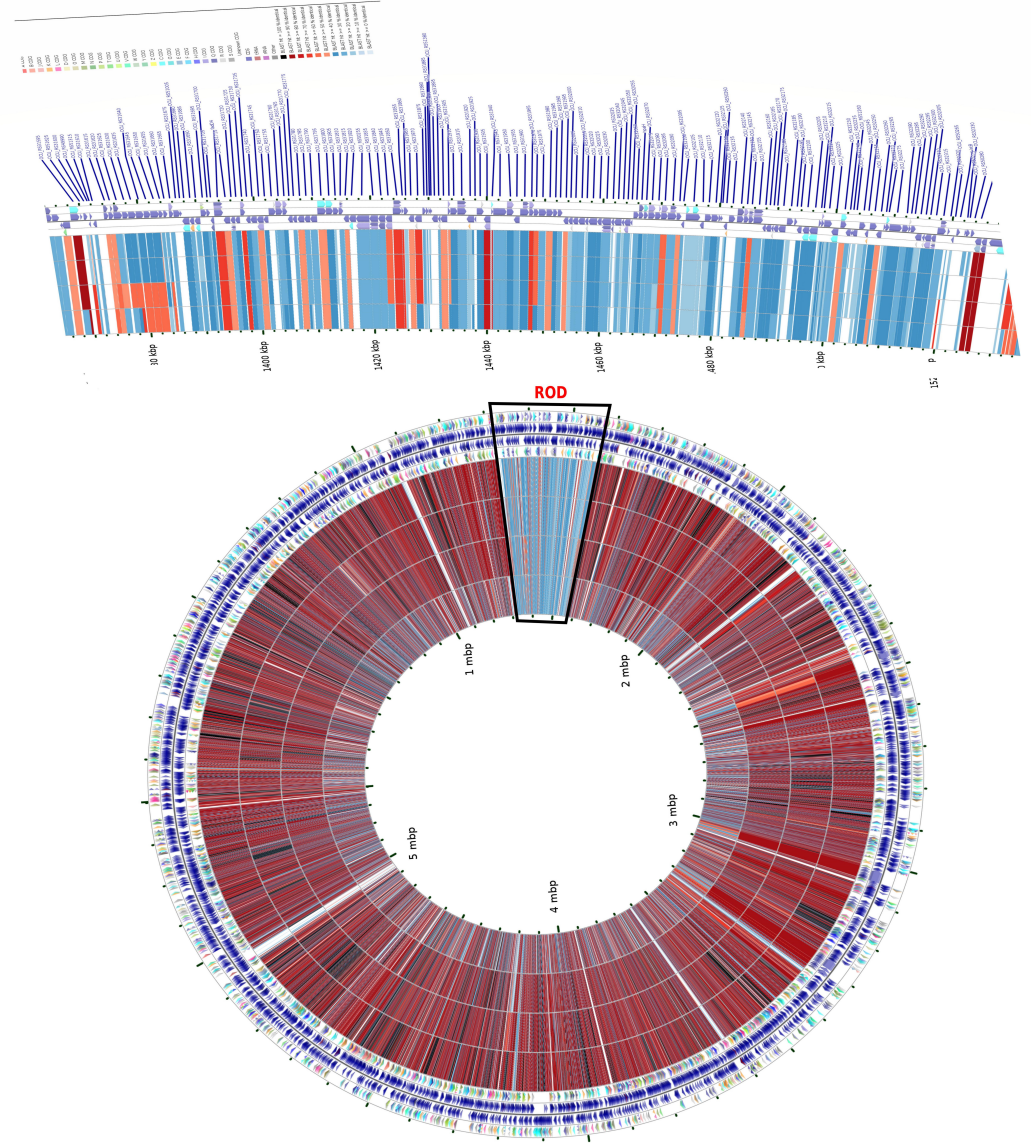
Genome-wide comparison of reference *M. intracellulare* ATCC 13950T genome with four intestinal *M. intracellulare* isolate genomes. CDS in forward and reverse directions for ATCC 13950T genome are indicated in the outer rings, and BLAST-based comparisons of *M. intracellulare* isolate genomes are presented as inner rings in order of highest to lowest similarity to ATCC 13950T genome. The innermost comparison ring begins with *M. intracellulare* C1, *M. intracellulare* S24, *M. intracellulare* A1, and *M. intracellulare* B1.

Kim et al. first reported the reference genome of *M. intracellulare* species (*M. intracellulare* Strain ATCC 13950T) (47). We mapped four intestinal *M. intracellulare* isolate genomes against reference *M. intracellulare* strain ATCC 13950T (Fig. 3). Approximately 110 kbp long ROD has been identified in all four intestinal isolates (Fig. 3). A close look at ROD revealed that 156 protein-coding genes were absent in isolate genomes in relation to the reference *M. intracellulare* strain. These absent genetic regions encompass genes vital for diverse functions, such as transporter activity, transcriptional regulation, and cellular metabolism (see Table S2 at https://doi.org/10.6084/m9.figshare.30356617.v1).

### Resistance, virulence, and pangenome of *M. intracellulare* strains

We observed four antibiotic resistance genes (rpob2, RbpA, efpA, and mtrA) consistently present in *M. intracellulare* genomes (see Fig. S1 at https://doi.org/10.6084/m9.figshare.30356617.v1). Three out of four isolate genomes showed rpob2, RbpA, efpA, and mtrA resistance, while the C1 isolate lacked rpob2 and RbpA genes, which may be due to its reduced genome size and relatively less CDS compared to other isolate genomes as described in Table 1 (see Table S3 at https://doi.org/10.6084/m9.figshare.30356617.v1). The absence of rpob2 identification in strain C1 is likely attributable to the lower assembly quality metrics observed for this strain, as indicated by lower completeness and higher contamination levels assessed by CheckM tool (Table 1). These antibiotic resistance genes mainly conferred resistance to rifampicin, isoniazid, and macrolide antibiotics (see Fig. S1 at https://doi.org/10.6084/m9.figshare.30356617.v1). Intriguingly, *M. intracellulare* strains encode multiple ubiquitous virulence factor genes. A total of 20 virulence factor genes were identified, esxM (ESX-5 type VII secretion system EsxA, ESAT-6) and esxN (ESX-5 type VII secretion system EsxA, ESAT-6) being the most frequently occurring virulence factor genes within *M. intracellulare* species (see Fig. S2 at https://doi.org/10.6084/m9.figshare.30356617.v1). The presence/absence profile of various virulence factor genes is depicted in color heatmap (see Fig. S2 at https://doi.org/10.6084/m9.figshare.30356617.v1). Additionally, variation of pan and core gene content among different *M. intracellulare* strains was assessed in this study. Pan and core gene analysis data were fitted to exponential models (48, 49) to plot core and pan genome size.

**Fig 4:**
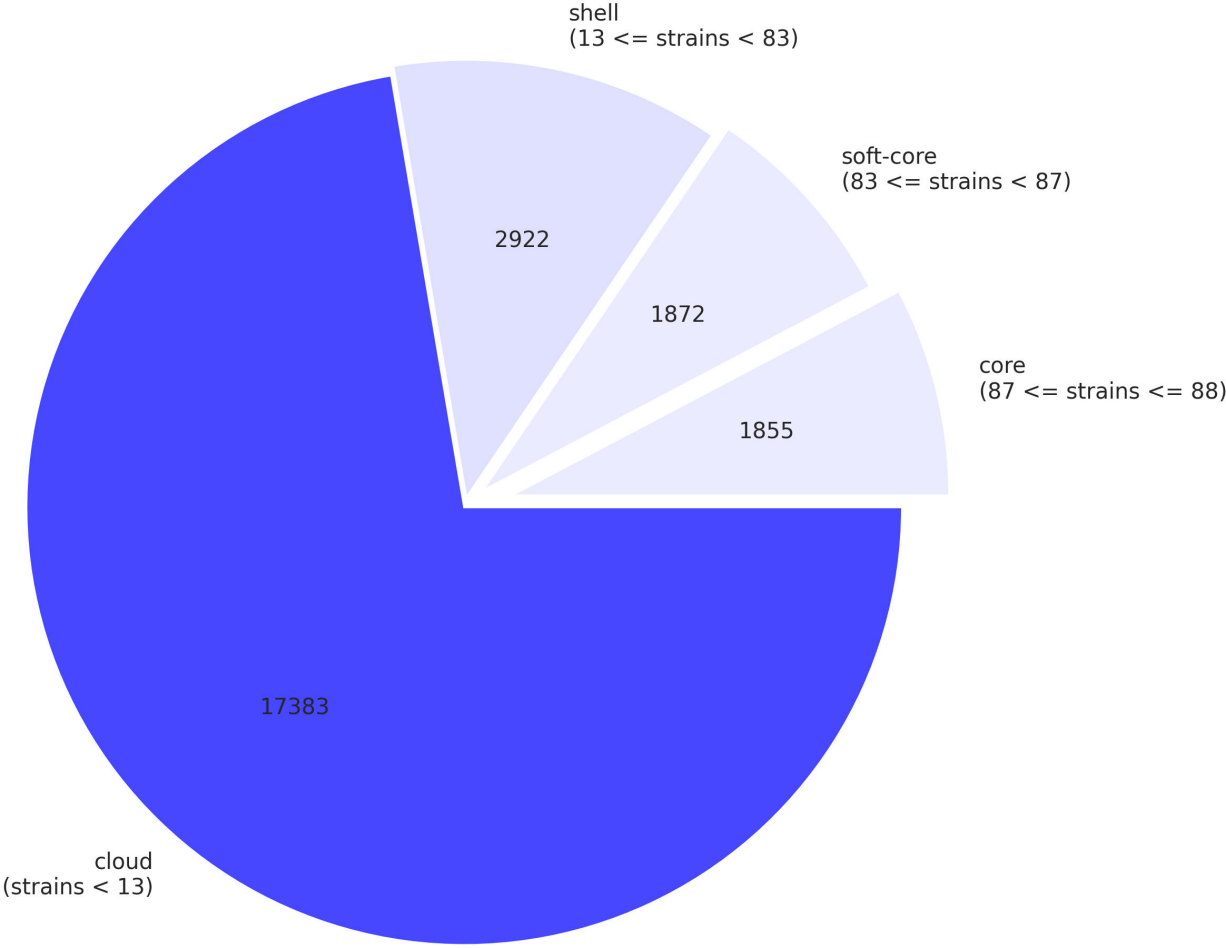
Pan genome profile of *M. intracellulare* strains.

A comprehensive pangenome analysis, combining four *M. intracellulare* strains along with 82 additional strains sourced from the NCBI database, identified 1,855 core genes, 1,872 soft-core genes, 2,922 shell genes, and 17,383 cloud genes (Fig. 4). Core genes are highly conserved and provide phylogenetic information, whereas shell and cloud genes are more flexible and vary across the genomes, demonstrating enhanced genomic adaptability and diversity (50). The core genome accounted for merely 7.71% of the entire pangenome, leaving the vast majority 92.29% as accessory genes. With the sampling of additional *M. intracellulare* genomes, an increase in the number of shell and cloud genes is anticipated (13). Certain *M. intracellulare* strains were observed to cluster into a distinct group on the pangenome phylogenetic tree, characterized by a relatively higher abundance of accessory genes (see Fig. S3 at https://doi.org/10.6084/m9.figshare.30356617.v1). This finding coincides with the high percentage of the flexible genome being previously observed in the pangenome analysis of 55 *M*. *intracellulare* genomes (13).

### Essential genes and recombination analysis

**Fig 5:**
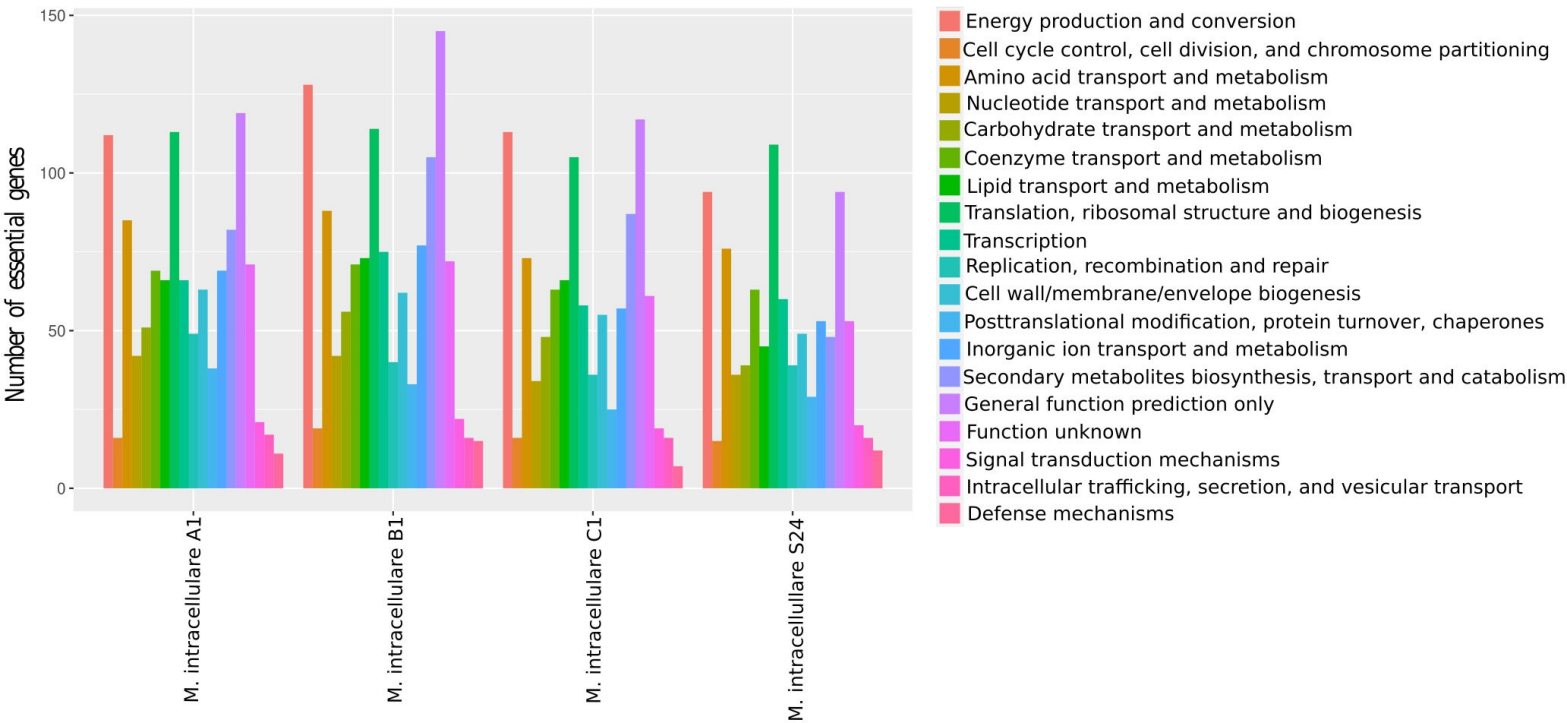
COG functional profile of essential genes predicted from intestinal *M. intracellulare* isolate genomes.

**Fig 6:**
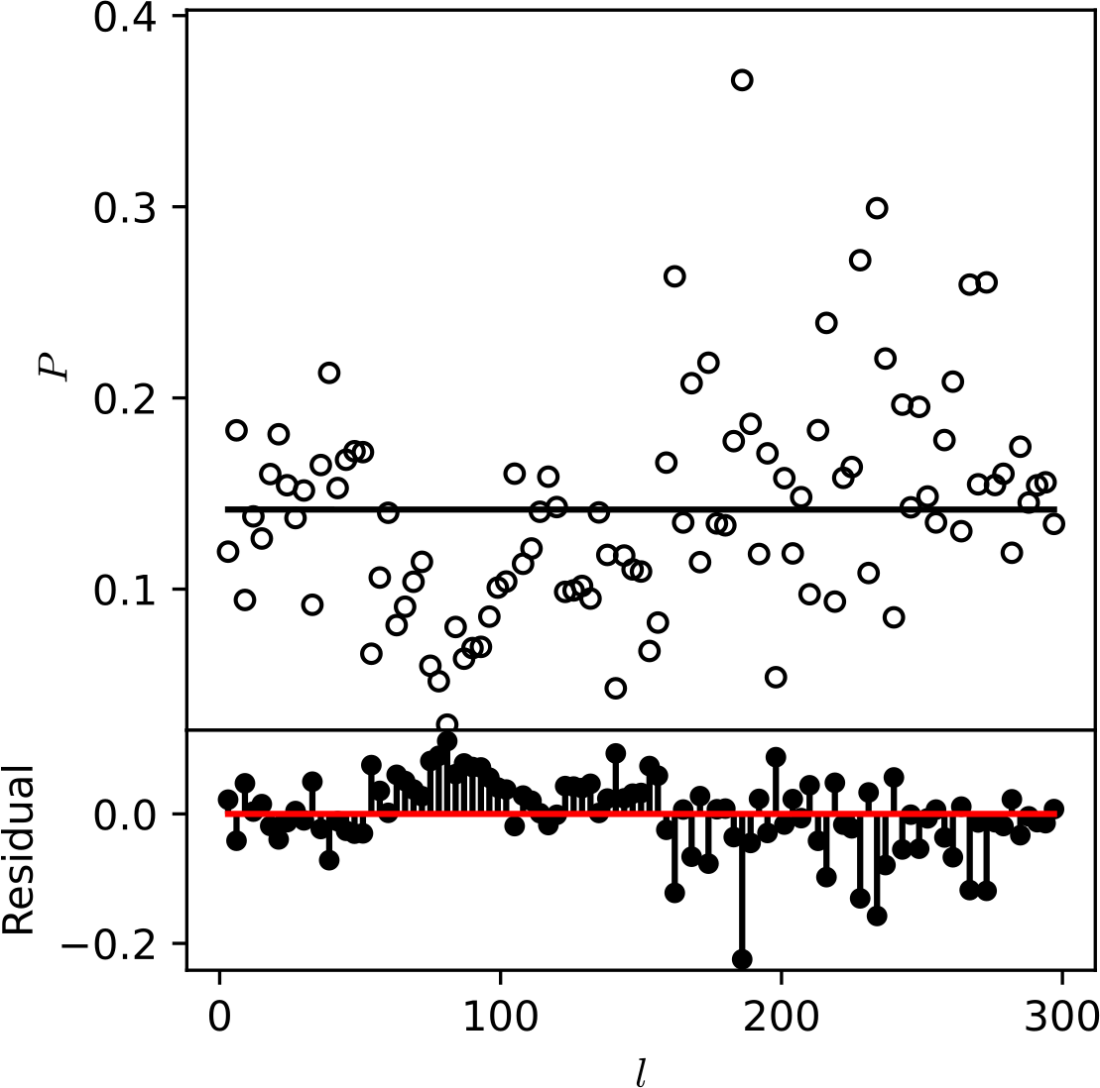
Correlation profiles of synonymous substitutions for the core genes in 86 *M*. *intracellulare* genomes.

Gene essentiality analysis predicted 1,426, 1,512, 1,300, and 1,141 essential genes in *M. intracellulare* isolates A1, B1, C1, and S24, respectively. Subsequently, functional profiling of essential genes unveiled a consistent abundance of certain COG functions (energy production and conversion, translation ribosomal structure and biogenesis, amino acid transport and metabolism, and general functions), which appeared to be essential for intestinal *M. intracellulare* isolates (Fig. 5). These COG metabolic functions might be crucial for maintaining the cellular processes of intestinal *M. intracellulare* strains. Over the last decade, significant progress has been made in understanding the phenomenon of “genome reduction syndrome” driven by close symbiotic relationships (51). To investigate genome reduction, we inquire into potential deletion-prone genomic regions. *M. intracellulare* S-24 harbors 27 deletion-prone segments, highest among all intestinal *M. intracellulare* isolates, constituting 17.3% of its entire genome, followed by *M. intracellulare* A1 (9 segments), *M. intracellulare* B1 (8 segments), and *M. intracellulare* C1 (2 segments) (see Table S4 at https://doi.org/10.6084/m9.figshare.30356617.v1). Additionally, we employed the mcorr method to investigate the rate of homologous recombination within *M. intracellulare* strains, which creates a correlation profile by comparing pairs of genomes to assess the probability of differences at one site given differences at another locus (35). From our analysis, we observed a flat correlation profile as a function of locus distance (*l*), indicating a weak recombination signal in *M. intracellulare* strains (Fig. 6) (35). Multiple recombination parameters including sample diversity (*d*), mutational divergence (*θ*), recombinational divergence (*ϕ*), relative rate of recombination to mutation (*γ*/*μ*), and recombination coverage (*c*) were obtained from the recombination analysis of *M. intracellulare* strains (see Table S5 at https://doi.org/10.6084/m9.figshare.30356617.v1). The *d* value, or sample diversity, in *M. intracellulare* species is estimated to be 0.10, derived from both recombination and mutations contributing to clonal evolution. This suggests that *M. intracellulare* exhibits greater sample diversity in comparison to other pathogenic bacterial species (*K. pneumoniae*, *M. abscessus*, *M. tuberculosis*, *P. aeruginosa*, *S. aureus,* and *Y. pestis*) (35). In *M. intracellulare* strains, the remaining four recombinant parameters identified as *θ*, *ϕ*, *γ*/*μ*, and *C* had values of 0.11, 0.01, 7.2, and 0.7, respectively. Mutational divergence (θ), defined as mean of mutation numbers per locus since the divergence of a pair of homologous sites, is found to have higher value for *M. intracellulare* compared to other pathogenic species such as *P. aeruginosa* (35, 52), *M. abscessus* (35, 53), *K. pneumoniae* (35, 54), *S. enterica* (55), and *S. pseudintermedius* (56). For *M. intracellulare* stains, the recombination divergence (*ϕ*) and recombination coverage (*c*) exhibited a lower value of 0.01 and 0.7 compared to other pathogenic bacterial species (35, 55, 56). Similarly, the relative rate of recombination to mutation (*γ*/*μ*) showed a lower value of 0.7% compared to *S. enterica* subsp. *enterica* (55) and *P. aeruginosa* (52), respectively.

## DISCUSSION

Multilocus sequence analysis has been extensively employed in phylogenomic studies to discern evolutionary signatures among MAC members influenced by environmental factors and selection pressures (3, 57). In this study, the core genome phylogenetic tree indicates that the evolution of *M. intracellulare* strains is independent of their isolation sources, as they clustered differently irrespective of their origins and host sources (Fig. 1). The functional heatmap distinctly categorized *M. intracellulare* genomes into five major groups, each showcasing a distinct functional profile. It is evident that the US (WUMAC) isolates including isolate S-24 (intestinal isolate) constitute Cluster 1, which exhibits reduced functional capability (Fig. 2). Upon careful analysis of the functional heatmap, it is apparent that there is a steady decline in functional capacity within *M. intracellulare* strains. Cluster 1 has shown the lowest metabolic capacity, followed by Cluster 5, Cluster 3, Cluster 4, and Cluster 2, respectively. It is worth noting that Cluster 1 *M*. *intracellulare* WUMAC strains obtained from patients with pulmonary MAC infections, which indicates host adaptation may lead to decreased metabolic functions of WUMAC strains (3). This observation highlights the importance of understanding and addressing the metabolic capacity of MAC isolates to get insights into the host adaptation of *Mycobacterium* species. Overall, it is plausible that *M. intracellulare* species is undergoing a genome streamlining (genome reduction) process, potentially making it more reliant on host machinery. It is important because, in addition to RODs, it provides further evidence supporting the presence of deletion-prone (dispensable) genomic regions in *M. intracellulare* isolates analyzed in this study (Fig. 3). Studies have reported deletions in *Mycobacterium* genomes, suggesting that these deletions could be mildly deleterious to the organisms, yet they may confer an advantage by aiding in evasion of the host immune system and thereby promoting transmission (58, 59). In intestinal *M. intracellulare* isolates, the dispensable genetic region does not involve genes exclusively related to pathogenicity or antibiotic resistance; it was collectively associated with energy metabolism essential for *in vivo* survival of bacteria (60). This provides insight into how *M. intracellulare* genomes are evolving, becoming increasingly dependent on host machinery by reducing metabolic genes while retaining the genes necessary for survival in the host environment. Horizontal gene transfer events contributed significantly to shape virulence properties, immune evasion, and adaptation of MAC species (61, 62). Comparative genomics revealed members of the MAC likely have an open pangenome (61, 62), which is consistent with our findings demonstrating the open pangenome of *M. intracellulare* strain (see Fig. S4 at https://doi.org/10.6084/m9.figshare.30356617.v1) (13). We investigated orthologous gene families in *M. intracellulare* pangenomes and found that members of sub-clade 2.1 exhibited an expanded pan-genome gene family (see Fig. S3 at https://doi.org/10.6084/m9.figshare.30356617.v1). This suggests that *M. intracellulare* strains, particularly those from sub-clade 2.1, may be undergoing significant variation in gene content and exhibiting increased genetic redundancy. The acquisition of exogenous genetic elements and the preferential retention of mobile genetic elements over non-essential genetic information may significantly contribute to the survival and evolutionary adaptability of the strains within this clade (63–65).

Antibiotic resistance gene analysis reveals that *M. intracellulare* strains do not exhibit a broad spectrum of antibiotic resistance, aligning with the findings of Fernández et al. (66). Furthermore, the overrepresentation of rifampicin and isoniazid resistance is consistent with the results reported by Mizuguchi et al. (67). Unlike antibiotic resistance genes, *M. intracellulare* species possess a higher number of virulence genes, supporting the potential virulence of human-associated *M. intracellulare* strains (68). The overrepresentation of esxN and esxM virulence genes suggests an active ESX-5 system in *M. intracellulare* strains. This could potentially explain the reduced cell wall integrity and enhanced virulence properties observed in *M. intracellulare* species (69).

The investigation of essential genes holds significant importance in various domains, such as synthetic biology, drug discovery, and vaccine development (70). Essential bacterial proteins have emerged as potential drug targets for new antibiotics owing to their critical role in bacterial life (71). To date, only one study, conducted by Tateishi et al. (18), investigated *M. intracellulare* essential genes using transposon sequencing. Tateishi et al. identified several genes related to anti-tuberculous drug targets, virulence factors, and carbon metabolism as crucial for the survival and functionality of *M. intracellulare* species (18). Our analysis indicates information storage and processing and metabolism is the most essential, followed by energy production and conversion, translation and ribosomal structure biogenesis, and amino acid transport and metabolism (Fig. 5). This result is consistent with earlier findings, suggesting that essential genes in bacterial species are primarily involved in functions related to metabolism, information storage, and processing (72–74). However, it should be noted that a significant portion (28–54%) of essential genes are poorly characterized or lack COG annotation. This may be due to the presence of an overwhelming number of 'conserved hypothetical' proteins that have become essential (75), thereby expanding the repertoire of essential hypothetical proteins in the *M. intracellulare* species group (76). Experimental studies have demonstrated that bacterial genomes exhibit preference for deletion over expansion events, and significant deletions can take place in a short evolutionary time frame (77–79). *M. intracellulare* isolate genomes were analyzed to predict deletion-prone or dispensable genomic regions. S24 strain exhibits the highest number of deletion-prone genomic regions, constituting approximately 17.3% of its total genomic regions, followed by A1 (5.6%), B1 (4.6%), and C1 (1.2%). Intestinal *M. intracellulare* strains harboring dispensable genomic regions highlight ongoing genomic reduction events that drive reduced genomic complexity (80), improved genomic stability, and optimized energy costs for cellular functions (81). Interestingly, this is congruent with the identification of RODs in four isolates, which are absent in comparison to the reference *M. intracellulare* strain (Fig. 3). These findings shed light on the diverse genomic features of *M. intracellulare* strains and their potential impact on survival and adaptation in various environments.

It is generally assumed that horizontal gene transfer and recombination are rare in NTM species; these phenomena are believed to have occurred mainly in their ancestral members during the early phases of the evolutionary process of the *M. tuberculosis* complex (MTBC) strains (82–84). In recent years, several genomic studies on MTBC members (85), with a focus on *M. bovis* (86), have highlighted the instances of recombination in MTBC strains. However, the limited nucleotide sequence variation often makes it difficult to detect these events. In this study, all publicly available *M. intracellulare* genomes (*n* = 86), along with intestinal isolate genomes (*n* = 4), were investigated to confirm any recombination events within the *M. intracellulare* species group. Recombination analysis using the mcorr pipeline unveiled a constant or flat correlation profile, indicating either a weak or complete absence of recombination signal within *M. intracellulare* strains (Fig. 6). We used five recombination and evolutionary parameters to better understand the recombination events. Sample diversity (*d*) and mutational divergence (*θ*) were observed highest within *M. intracellulare* species compared to other pathogens. However, the rest of the recombination parameters (*ϕ*, *γ*/*μ*, and *c*) had lower values, suggesting a low recombination rate, lower genomic heterogeneity, and clonal evolution of *M. intracellulare* strains. The globally distributed *M. intracellulare* strains might have undergone significant ecological and geographical constraints, leading to a well-defined population structure that limits gene flow among members, resulting in fewer or no recombination events within the *M. intracellulare* population (87). High mutational divergence supports the notion that mutations may have played a major role in driving sequence diversity in *M. intracellulare* strains. This finding further corroborates earlier reports suggesting higher mutation rates among members of the MAC (3, 88).

### Conclusion

Our study revealed a high genetic diversity of intestinal *M. intracellulare* strains isolated from IBD patients in India. The existence of deletion-prone genomic regions suggests underlying genome streamlining processes, potentially shifting the metabolic capacity of intestinal strains and transforming them into more dependent on host machinery. Isolation source, host ecology, and recombination are loosely associated with the genetic evolution of *M. intracellulare* strains. Genome reduction through reductive evolution might have a greater impact on the genome evolution of the intestinal *M. intracellulare* isolate. Essential genes were mainly involved in key cellular functions and are critical to design drug targets to fight against *M. intracellulare* infection. Further research is necessary to better understand the biology of *M. intracellulare* strains, particularly poorly characterized essential genes.

## Data Availability

The data sets generated in this study are deposited in the NCBI with BioProject accession number PRJNA992578. Whole-genome assemblies are deposited in GenBank under the accession numbers JAUZTI010000000, JAUZTJ010000000, JAUZTK010000000, and JAKFBO010000000, respectively. Supplementary files are available at: https://doi.org/10.6084/m9.figshare.30356617.v1.
